# Myositis as an Extraintestinal Manifestation of Ulcerative Colitis: A Case Report and Literature Review

**DOI:** 10.7759/cureus.42336

**Published:** 2023-07-23

**Authors:** Talwinder K Nagi, Yousra Gheit, Oscar L Hernandez, Zoilo K Suarez, Charles Vallejo, Muhammad Adnan Haider, Touqir Zahra

**Affiliations:** 1 Internal Medicine, Florida Atlantic University Charles E. Schmidt College of Medicine, Boca Raton, USA; 2 Medicine, Florida Atlantic University Charles E. Schmidt College of Medicine, Boca Raton, USA; 3 Internal Medicine, Florida Atlantic University, Boca Raton, USA

**Keywords:** extraintestinal manifestations, crohn’s, dermatomyositis, polymyositis myalgia, inflammatory myositis, ulcerative colitis (uc)

## Abstract

Ulcerative colitis (UC) is an inflammatory bowel disease (IBD) that is thoroughly studied and known to have a strong genetic component. It affects the mucosa and submucosa of the colon and rectum, causing diffuse friability and superficial erosions leading to bleeding. Common presenting symptoms include diarrhea that is often bloody or purulent and abdominal pain or cramping. There are also extraintestinal manifestations of UC such as cutaneous rashes, eye inflammation, and oral ulceration. A rarer manifestation of IBD is myositis, either dermatomyositis, polymyositis, or even rhabdomyolysis. Based on the literature review, myositis has been documented more so in cases of Crohn’s disease versus UC. In this report, we discuss a patient with known UC who presented during a flare and subsequently complained of diffuse myalgia. She was found to have an elevated creatine kinase (CK), thus suggesting some form of myositis. We will review possible pathogenesis and other cases of UC presenting with myositis that have been documented.

## Introduction

Ulcerative colitis (UC) is a chronic systemic inflammatory condition affecting the large bowel mucosa and can present intermittently with extraintestinal symptoms. Although there is no specific cause, there may be a strong genetic component predisposing individuals to the development of UC. The pathophysiology involves defects in the epithelial barrier and leukocyte recruitment and within the microflora of the colon. Primarily, the epithelial barrier may have faulty colonic mucin and tight junctions that increase the uptake of luminal antigens. Leukocyte recruitment is upregulated due to an increase of the chemoattractant CXCL8 and mucosal vascular addressin cell adhesion molecule 1 (MadCAM1), which causes leukocyte adhesion and extravasation into the mucosal tissue. Although the pathogenesis is not fully understood, the development of UC could be attributed to an imbalance between enteric microflora in mucosal immunity, causing the individual to respond to nonpathogenic bacteria found in their intestines [1-5].

In addition to the gastrointestinal symptoms of diarrhea, abdominal pain, and hematochezia, other manifestations may include arthritis, uveitis, scleritis, anemia, and various skin findings such as pyoderma gangrenosum. Rarely, skeletal muscle involvement may also occur in the patients with inflammatory bowel disease (IBD). It is more closely associated with Crohn’s disease, as only a few cases worldwide have been reported with UC [6-8].

In this report, we present a patient treated for a UC flare who subsequently developed symptoms of muscle weakness and pain, suggesting myositis. We will review other reports documented in the literature and discuss a possible mechanism linking the development of myositis in the patients with UC.

## Case presentation

A 75-year-old female with a medical history of ulcerative colitis (UC), type 2 diabetes mellitus, and hypothyroidism presented to the emergency department due to progressive bloody and mucoid diarrhea for three weeks with abdominal pain. The diarrhea would occur 5-10 times per day and was associated with tenesmus. The tenesmus pain would start diffusely in the epigastric region and was described as a burning sensation, which would radiate to her bilateral lower abdomen during bowel movements. The pain was moderately relieved after completing a bowel movement. She endorsed mild nausea and clear vomiting during the abdominal discomfort, which started five days prior. She had been taking loperamide, which helped reduce the frequency of bowel movements. Per the patient, she has a flare of her UC once a year and follows closely with her outpatient gastroenterologist. She had been compliant with her prescribed mesalamine for which she takes two tablets of a 1.2 g dose twice daily. She denied any feelings of fever or chills, recent travel history or possible sick contact, and recent antibiotic use. She has no history of smoking or illicit drug use and only drank a maximum of two glasses of wine per day. Of note, the patient mentioned that the nausea and vomiting are usually not associated with her UC flares and that the abdominal pain feels different from prior pain during flares.

**Figure 1:**
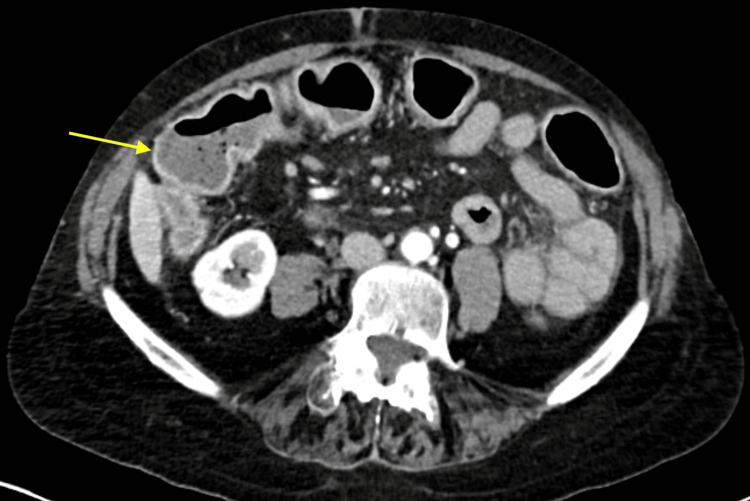
CT scan of the abdomen and pelvis with contrast depicting pancolitis with wall thickening CT: computed tomography

**Figure 2:**
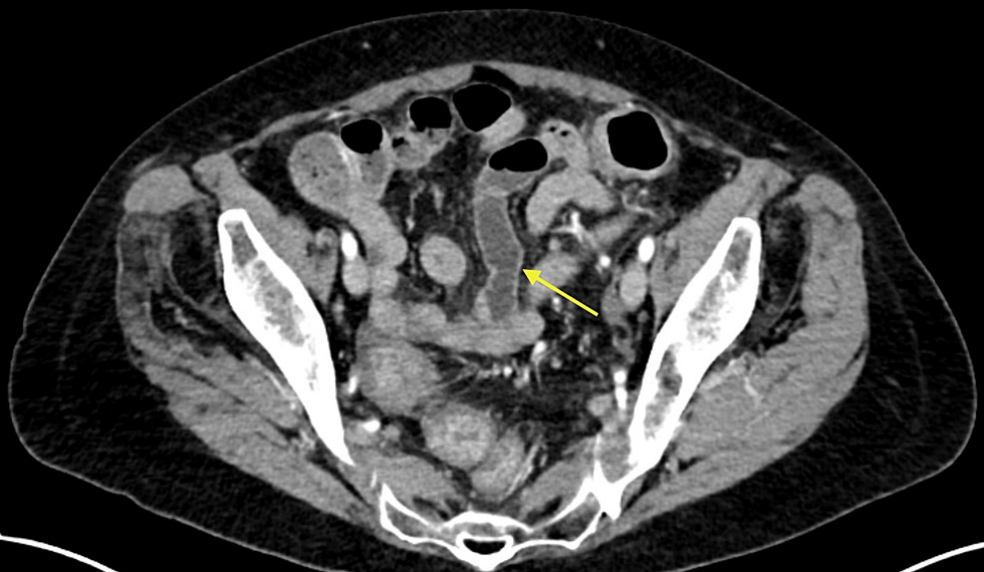
CT scan of the abdomen and pelvis with contrast depicting pancolitis with wall thickening CT: computed tomography

**Figure 3:**
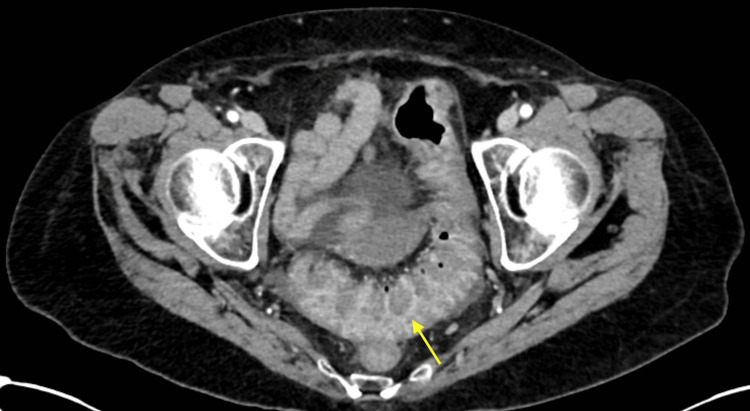
CT scan of the abdomen and pelvis with contrast depicting pancolitis with wall thickening CT: computed tomography

On presentation, vitals were hemodynamically stable, and she was not febrile. On examination, the only pertinent finding was tenderness to palpation of the lower abdominal quadrants. Laboratory evaluation was significant for an elevated white blood cell count of 10.1 K/mcL, hemoglobin of 11.7 g/dL with a hematocrit of 35.9%, and aspartate aminotransferase (AST) and alanine aminotransferase (ALT) elevated to 82 IU/L and 69 IU/L, respectively. A right upper quadrant ultrasound was completed due to the symptom of tenesmus starting in the epigastric region. The result was unremarkable with a normal common bile duct measuring 0.7 cm and a normal-appearing gallbladder. A computed tomography (CT) scan of the abdomen and pelvis with contrast revealed pancolitis with wall thickening involving the rectum and mild diffuse hepatic steatosis as depicted in Figures 1-3. Stool studies for *Salmonella*, *Shigella*, *Campylobacter*, Shiga toxin, and *Clostridium difficile* were negative. She was continued on her home mesalamine dose with the initiation of intravenous methylprednisolone 40 mg every eight hours for the management of colitis.

The patient began to show symptomatic improvement in abdominal pain with less frequent diarrhea. On day 3 of admission, laboratory results were significant for an elevated AST of 354 IU/L and ALT of 150 IU/L and fecal calprotectin of 437 mcg/g. She began to complain of worsening diffuse body aches and darker-colored urine. A hepatitis panel and creatine kinase (CK) level were checked. She experienced mild pain during the range of motion of her upper and lower extremities. The results for the hepatitis panel were unremarkable; however, CK was elevated to 7,440 IU/L; thus, intravenous lactated Ringer fluid was started. Transaminitis was thought to be secondary to the elevated CK, and after the initiation of the fluids, the levels began to downtrend. The patient continued to deny further abdominal pain or diarrhea, the methylprednisolone was discontinued, and prednisone 40 mg daily was initiated. The patient was ultimately deemed stable for discharge with the resolution of her body aches and clear-yellow-appearing urine. The AST and ALT on discharge were 173 IU/L and 179 IU/L, respectively, and CK was 1,397 IU/L. She continued on her home medications with a prednisone taper prescribed and will continue with close outpatient follow-up with her gastroenterologist.

## Discussion

Myositis is a rare extraintestinal manifestation of IBD initially reported in 1970. Based on a literature review through PubMed, it has been more commonly reported in flares of Crohn’s disease versus UC. Data gathered from published case reports have been summarized in Table 1. The most common myositis documented is polymyositis compared to dermatomyositis. The mainstay of diagnosis and treatment is a muscle biopsy and steroids, respectively [9]. Unfortunately in our case, a muscle biopsy was not completed, which could have further supported the diagnosis. However, other etiologies of the patient’s myositis were considered but did not account for the presenting symptoms and clinical course. Despite the cases that have already been documented, the understanding and knowledge of this extraintestinal manifestation are not well known.

**Table 1:** Literature on case reports gathered through PubMed using the keywords “myositis” and “ulcerative colitis” from 1986 to 2022 M, male; F, female; Sx, symptoms; Dx, diagnosis; UC, ulcerative colitis; PM, polymyositis; DM, dermatomyositis; EMG, electromyography; CT, computed tomography, IVIG, intravenous immunoglobulin

Author	Age	Sex	Myositis	Sx of myositis	Dx modality	Treatment	UC status
Bhigjee et al. (1987) [10]	44	M	Interstitial myositis	Weakness	Muscle biopsy	Steroid and azathioprine	Dormant
Evrard et al. (1987) [11]	33	M	PM	Pain, swelling, and fever	Muscle biopsy	Steroid and sulfasalazine	Active
Kaneoka et al. (1990) [12]	57	F	PM	Weakness	Muscle biopsy	Steroid	Dormant
Chugh et al. (1993) [2]	78	F	PM	Proximal muscle weakness, upper and lower limbs	Muscle biopsy	Steroid	Active
Voigt et al. (1999) [13]	33	F	PM	Pain, weakness, and fever	EMG and MRI (biopsy refused)	Steroid	Dormant
Qureshi et al. (2002) [8]	36	M	Neutrophilic myositis	Rash, pain, and swelling	Muscle biopsy	Steroid	Dormant
Paoluzi et al. (2006) [14]	51	F	PM	Pain, weakness, and fever	EMG	Steroid	Active
González García et al. (2016) [15]	58	M	PM	Swelling and weakness	Muscle biopsy	Steroid and methotrexate	Active
Jain and Gottlob (2001) [16]	43	F	PM	Diplopia and ocular pain	CT	Steroid	Dormant
Naramala et al. (2019) [17]	28	M	PM	Myalgia of bilateral lower extremities	MRI and muscle biopsy	Steroid	Dormant
Kim et al. (2020) [1]	14	F	PM	Pain and swelling	MRI and muscle biopsy	Steroid, cyclosporine, and IVIG	Active
Huang et al. (2020) [18]	57	F	DM	Lower extremity weakness	Muscle biopsy	Infliximab	Active

One case from India presented a 78-year-old female with chronic bloody diarrhea for 15 years, who presented with a new complaint of diffuse arthralgias involving multiple joints. A colonoscopy confirmed the diagnosis of UC, and a muscle biopsy from the left quadricep revealed focal intermyseal infiltration with lymphocytes. She had no prior history of muscle injury or myositis. Her symptoms subsequently resolved with 5-acetylsalicylic acid and prednisone after 12 weeks of therapy [2].

Myositis has also been documented to even precede gastrointestinal symptoms of UC. In a pediatric case, a 14-year-old female presented with severe shoulder and left mandible pain that started three days prior to hematochezia, abdominal pain, vomiting, and diarrhea. An MRI had revealed myositis in the deltoid and teres minor muscles, while a colonoscopy confirmed the diagnosis of UC. Myositis in the setting of UC was confirmed with a muscle biopsy showing degeneration, atrophy, and necrosis. The patient was managed with steroids, mesalamine, and azathioprine. Cyclosporine and intravenous immunoglobulin (IVIG) were added for steroid-resistant pyoderma gangrenosum [1].

The mechanism of myositis developing during or even preceding UC remains unknown. Genetic factors such as interferon regulatory factor 5 (IRF5) rs4728142 and the vitamin D receptor rs2228570 have been thought to play a role in this association [8-10]. However, there is an immune-mediated hypothesis proposing that bowel inflammation and mucosal damage lead to the release of antigens, which stimulate an immune response. Subsequently, these intestinal antigens induce antibody-forming immune complexes that can ultimately result in muscle injury [5,6]. Another possible mechanism suggests that myositis is due to an immune response initiated by an infectious agent such as the measles virus, herpes virus, paramyxovirus, or mycobacterium paratuberculosis present in the large bowel and striated muscles. These organisms have been implicated in the pathogenesis of IBD and myositis. Finally, some authors have thought that therapy for IBD, including glucocorticoids or azathioprine, may be responsible for muscle injury. However, in this circumstance, inflammatory myopathy could be differentiated from steroid myopathy through a muscle biopsy [1-6].

## Conclusions

In a patient presenting with likely UC flare complaining of myalgia or similar complaints in a patient with known IBD, myositis as an extraintestinal manifestation should be high on the differential. A CK level should be assessed to help with diagnosis and management, especially if elevated enough to suggest rhabdomyolysis. We highly emphasize the necessity of completing a muscle biopsy in order to further support the diagnosis, which was a limitation in our case and a learning point. Generally, these patients tend to improve with the treatment of underlying IBD based on the studies presented. Regardless, providers should be wary of myositis as an extraintestinal manifestation with new-onset IBD, in acute exacerbations, or even during remission.
